# Association of mineral metabolism biomarkers with chronic kidney disease in Chinese adults

**DOI:** 10.1007/s10157-021-02037-4

**Published:** 2021-03-16

**Authors:** Jialin Li, Danni He, Wenjing Zhao, Xi’ai Wu, Minjing Luo, Ying Wang, Meihua Yan, Wenquan Niu, Ping Li

**Affiliations:** 1 Beijing Key Lab Immune-Mediated Inflammatory Diseases, Institute of Clinical Medical Science, China-Japan Friendship Hospital, Beijing, China; 2 Department of Nephrology, Beijing Hospital of Traditional Chinese Medicine, Capital Medical University, Beijing, China; 3 Institute of Clinical Medical Sciences, China-Japan Friendship Hospital, Beijing, China; 4 Department of Endocrinology, China-Japan Friendship Hospital, Beijing, China

**Keywords:** Chronic kidney disease, Interaction, Mineral metabolism biomarkers, Propensity score matching, Risk and severity

## Abstract

**Background:**

We aimed to examine the association of three mineral metabolism markers, including serum calcium, inorganic phosphorus, and intact parathyroid hormone with the risk of chronic kidney disease (CKD) at all stages.

**Methods:**

This retrospective cohort study involved 3563 participants, including 3274 CKD patients and 289 healthy controls. CKD is diagnosed according to clinical guidelines from the 2012 KDIGO. Effect sizes are expressed odds ratio (OR) and 95 confidence interval (CI).

**Results:**

After propensity score matching, per 0.5 mg/dL increment of inorganic phosphorus was significantly associated with 1.33-, 1.61-, and 2.85-fold increased risk of CKD at stages 1–2, 4, and 5, respectively. Regarding per 8 pg/mL increment of intact parathyroid hormone, significance was only noted for stage 5. In subsidiary analyses, the risk prediction of mineral metabolism markers under study was more evident in males and hypertensive subjects. A nomogram prediction model was constructed based on age, sex, and three mineral metabolism markers for CKD, with decent accuracy.

**Conclusions:**

Our findings indicate that serum calcium was associated with all-stage CKD risk, whereas the association for inorganic phosphorus and intact parathyroid hormone was significant at advanced stages.

## Introduction

Chronic kidney disease constitutes a worldwide health problem due to its high prevalence and absolute burden. The global statistics show that the prevalence of chronic kidney disease is estimated to be 8–16% [1, 2]. In China, chronic kidney disease is a leading cause of death, and it affects approximately 10.8% of adults [3, 4]. It is imperative to curb the epidemic of chronic kidney disease by characterizing the prodromal features of chronic kidney disease at different stages [5].

It is well known that chronic kidney disease is complicated by the presence of abnormalities that reflect disruption in the excretory, metabolic, and endocrine function of the kidney, leading to a dysfunction of mineral metabolism [6–8]. Evidence from clinical and epidemiologic studies indicates that abnormalities of mineral metabolism, such as secondary hyperparathyroidism, hyperphosphatemia, and hypocalcemia, are commonly seen in patients with kidney failure and are associated with an increased risk for Chronic kidney disease–Mineral and Bone Disorder (CKD–MBD), vascular calcification, and cardiovascular events [9–11]. Some researchers reported that some mineral metabolism markers maintained within normal laboratory ranges until chronic kidney disease is advanced [12–14]. Currently, a majority of clinical investigations focused on the changes in phosphorus, calcium, and parathyroid hormones mainly in patients with stages 3–5 chronic kidney disease or dialysis patients [15–17]. Available data on the association of mineral metabolism markers with early-stage chronic kidney diseases are particularly sparse in the medical literature.

To fill this research gap and yield more information, we in a large retrospective cohort study aimed to examine the association of three mineral metabolism markers, including serum calcium (corrected by serum albumin, the same below), inorganic phosphorus, and intact parathyroid hormone with the risk of chronic kidney disease at all stages among Chinese adults, especially at early-stage. To facilitate clinical application, we constructed a nomogram prediction model of the severity of CKD based on significant attributes.

## Methods

### Study subjects

This study involved a total of 5294 subjects 18–80 years of age who were retrospectively recruited from Department of Endocrinology and Department of Nephrology at China–Japan Friendship Hospital during the period between January 2010–December 2018. The conduct of this study received ethical approval from China-Japan Friendship Hospital Ethics Committee and conformed to the principles of the Declaration of HELSINKI. All study subjects provided informed consent prior to participation.

### Eligible criteria

Among 5294 initially recruited subjects in this study, 1731 were excluded from the analysis because of specific reasons listed below: (i) reporting of receiving dialysis or kidney transplantation; (ii) missing data on serum creatinine and urinary albumin-to-creatinine ratio; (iii) use of oral drugs affecting mineral metabolism, such as Lanthanum carbonate, aluminum binders, calcium salts, Calcitriol or vitamin D; (iv) women in pregnancy; (v) diagnosis of diabetic ketoacidosis, acute cardiovascular events, or other severe disorders including tumors. So, the present analysis involved 3563 study subjects who had complete data.

### Sample size

Out of 3563 qualified subjects, 3274 were confirmed to have CKD at all stages, and they formed the case group. The remaining 289 subjects who had no clinical signs of CKD were in the control group.

### CKD diagnosis

CKD is diagnosed if estimated glomerular filtration rate is less than 60 ml/min/1.73 m^2^ or albuminuria is presented based on the clinical practice guidelines from the Kidney Disease Improving Global Outcomes (KDIGO) in 2012 [18]. Glomerular filtration rate is calculated according to the 2009 CKD Epidemiology Collaboration (CKD-EPI) equation, which is gender-specific and was confirmed by many previous reports [19–23].

Because most patients with CKD had proteinuria measured only one time, persistent proteinuria was not defined in this study.

### CKD stages

According to the KDIGO guidelines in 2012, patients with CKD were divided into four groups, that is, stage 1–2, stage 3, stage 4, and stage 5 [18].

In this present study, 471, 544, 1214, and 1045 patients were diagnosed to have CKD at stage 1–2, stage 3, stage 4, and stage 5, respectively.

### Clinical and biochemical markers

Besides age and sex, hypertension and diabetes mellitus were diagnosed at the time of recruitment. Hypertension was defined as systolic blood pressure ≥ 140 mmHg, diastolic blood pressure ≥ 90 mmHg, or the current use of antihypertensive agents [24]. Diabetes mellitus was defined as fasting plasma glucose ≥ 7.0 mmol/L or taking hypoglycemic drugs or receiving parenteral insulin therapy [25].

Venous blood was taken in the fasting state. The reference ranges of serum calcium, inorganic phosphorus, and intact parathyroid hormone are 2.51–5.52 mg/dL, 8.0–11 mg/dL, and 12–88 pg/mL, respectively. All assays were conducted at the Clinical Laboratory for Endocrinology, China–Japan Friendship Institute of Clinical Medical Sciences.

### Statistical analyses

To assess whether baseline characteristics differed significantly across cases with CKD at different stages and the controls, the *χ*^*2*^ test for categorical variables and Wilcoxon rank sum test for continuous variables were used. To assess the association of mineral metabolism markers with the risk and severity of CKD, Logistic regression analysis was performed before and after adjusting for covariates. To allow for balancing of covariates between cases and controls, propensity score matching on age, gender, diabetes, and hypertension was conducted. Risk magnitude is weighted using odds ratio (OR) and its 95% confidence interval (95% CI).

To assess prediction accuracy after adding each mineral metabolism marker to the basic model, net reclassification improvement and integrated discrimination improvement [26, 27] were calculated to judge the discrimination capability of significant mineral metabolism markers. Calibration capability was assessed using the -2 log likelihood ratio test, Akaike information criterion, and Bayesian information criterion to inspect how closely the prediction probability for the addition of significant mineral metabolism markers reflected the actual observed risk and the global fit of modified risk model.

To illustrate the interaction between mineral metabolism markers, the “rgl” package in the R Project for Statistical Computing (available at the website https://www.r-project.org/) was adopted to plot three-dimension surface. The core of “rgl” package is a shared library that acts as an interface between R and OpenGL.

The STATA software Release 14.1 (StataCorp, TX, USA) was used for data cleaning and statistical analyses unless otherwise indicated [28]. Statistics were adjusted for multiple testing using a Bonferroni correction. Without prior notice, statistical significance was set at a probability of less than 5%.

## Results

### Baseline characteristics

Table 1 shows the baseline characteristics of study subjects in this study. Cases with stages 1–4 CKD were older than controls, while cases with stage 5 were younger. Sex differed significantly between cases and controls, except for stage 3a and stage 3b CKD. Percentages of hypertension and diabetes mellitus were higher in cases with stages 4–5 CKD than in controls.

**Table 1:** Baseline characteristics of study subjects in this study

Characteristics	Controls		Cases with chronic kidney disease			
		Stage 1–2	Stage 3a	Stage 3b	Stage 4	Stage 5
Sample size	289	471	172	372	1214	1045
Age (years)	62 (53–69)	68 (56–75)^**^	71 (64–77)^**^	73(66–78)^**^	70 (60–76)^**^	59 (47–68)^**^
Male (*n*, %)	236, 81.7	325, 69^**^	126, 73.2^**^	302, 81.1^**^	819, 67.5^**^	(588, 56.3)^**^
Hypertension (*n*, %)	175, 60.6	308, 65.4	110, 64.0	244, 65.6	875, 72.1^**^	(821, 78.36)^**^
Diabetes (*n*, %)	111, 38.4	179, 38	66, 38.4	145, 39	544, 44.8^*^	356, 34.1
TG (mmol/L)	1.38 (1.00–1.90)	1.320 (0.94–2.03)	1.34 (0.93–1.96)	1.37 (0.95–1.95)	1.51 (1.05–2.20)^**^	1.54 (1.08–2.19)^**^
TC (mmol/L)	4.30 (3.52–4.96)	4.02 (3.08–4.80)^**^	3.69 (2.93–4.52)^**^	3.73 (2.96–4.58)^**^	3.95 (3.16–4.94)^**^	4.180 (3.45–4.99)
LDL-C (mmol/L)	2.56 (1.98–3.08)	2.29 (1.59–2.92)^**^	2.09 (1.48–2.73)^**^	2.12 (1.50–2.76)^**^	2.29 (1.64–3.06)^**^	2.42 (1.88–3.09)
HDL-C (mmol/L)	1.01 (0.84–1.21)	0.97 (0.72–1.22)^*^	0.87 (0.63–1.09)^**^	0.85 (0.61–1.06)^**^	0.91 (0.68–1.17)^**^	1.00 (0.78–1.24)
HbA1c (%)	6.10 (5.50–7.10)	6.20 (5.50–7.20)	6.30 (5.70–7.20)^*^	6.60 (5.90–7.40)^*^	6.40 (5.70–7.30)^*^	5.90 (5.30–6.70)^**^
UA (μmol/L)	333 (285–387)	314 (218–444)	379 (270–500)^**^	390 (278–520)^**^	442 (345–548)^**^	404 (316–502)^**^
SCr (μmol/L)	75.03 (66.10–85)	77.40 (62.90–92.65)	120.8 (110–168)^**^	169 (138–189)^**^	236 (203.3–283)^**^	635.9(463.6–859.0)^**^
ACR (mg/g)	5.70 (4.20–9.10)	64.80 (44.10–80.30)^**^	19.90 (6.60–32.10)^**^	23.70 (19.50–82.10)^**^	126.9 (36.1–317.3)^**^	220.6 (126.3–429.5)^**^
BUN (mmol/L)	5.44 (4.46–6.75)	9.07 (5.72–14.88)^**^	9.65 (7.83–13.76)^**^	13.65 (10.76–17.83)^**^	15.20 (11.83–20.12)^**^	21.24 (16.10–27.36)^**^
Ca (mg/dL)	8.88 (8.56–9.2)	8.44 (7.86–8.96)^**^	8.41 (7.92–8.91)^**^	8.40 (7.90–8.83)^**^	8.48 (7.88–8.96)^**^	8.44 (7.8–9.16)^**^
IP (mg/dL)	3.44 (3.10–3.84)	3.41 (2.69–4.15)	3.42 (2.93–4.09)	3.37 (2.65–4.03)	3.94 (3.29–4.65)^**^	5.05 (4.06–6.26)^**^
i-PTH (pg/mL)	34.3 (28.6–53.7)	86.55 (39.95–171.5)^**^	60.6 (38.2–96.4)^**^	65(40.2- 99.7)^**^	82.05 (45–130)^**^	174.6 (72.20–333) ^**^

*P* values are calculated by Wilcoxon rank sum tests for continuous variables expressed as median (interquartile range) and *χ*^*2*^ tests for categorical variables expressed as count and percent

*TG* triglycerides, *TC* total cholesterol, *LDL-C* low-density lipoprotein cholesterol, *HDL-C* high-density lipoprotein cholesterol, *HbA1c* hemoglobin A1c, *UA* uric acid, *Scr* serum creatinine, *ACR* albumin-to-creatinine ratio, *BUN* blood urea nitrogen, *IP* inorganic phosphorus, *Ca* serum calcium, *i-PTH* intact parathyroid hormone

^*^P < 0.05

^**^P < 0.01

**Fig. 1:**
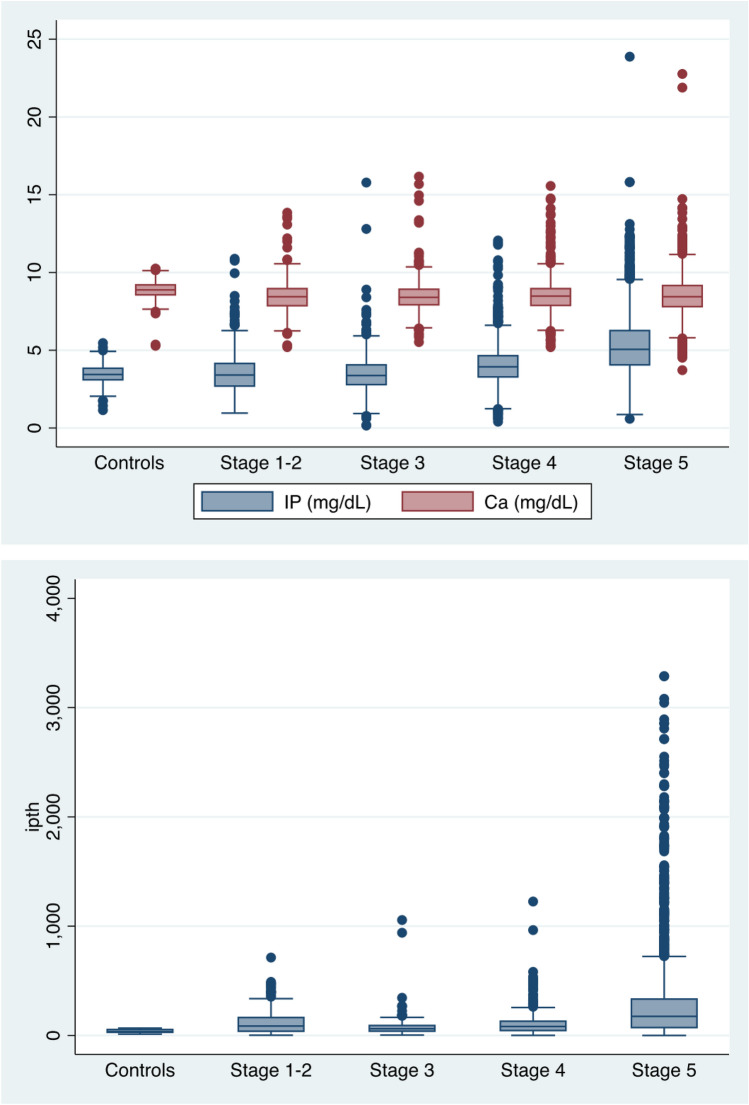
Distributions of serum inorganic phosphorus (IP), serum calcium (Ca), and intact parathyroid hormone (i-PTH) in both cases and controls

Figure 1 displays the distributions of three mineral metabolism markers in both cases and controls. Serum calcium concentrations were consistently lower in cases with CKD at all stages than controls, yet the concentrations of intact parathyroid hormone were consistently higher. For inorganic phosphorus, only cases with stages 4–5 CKD had higher concentrations than controls.

### Correlation analyses

**Fig. 2:**
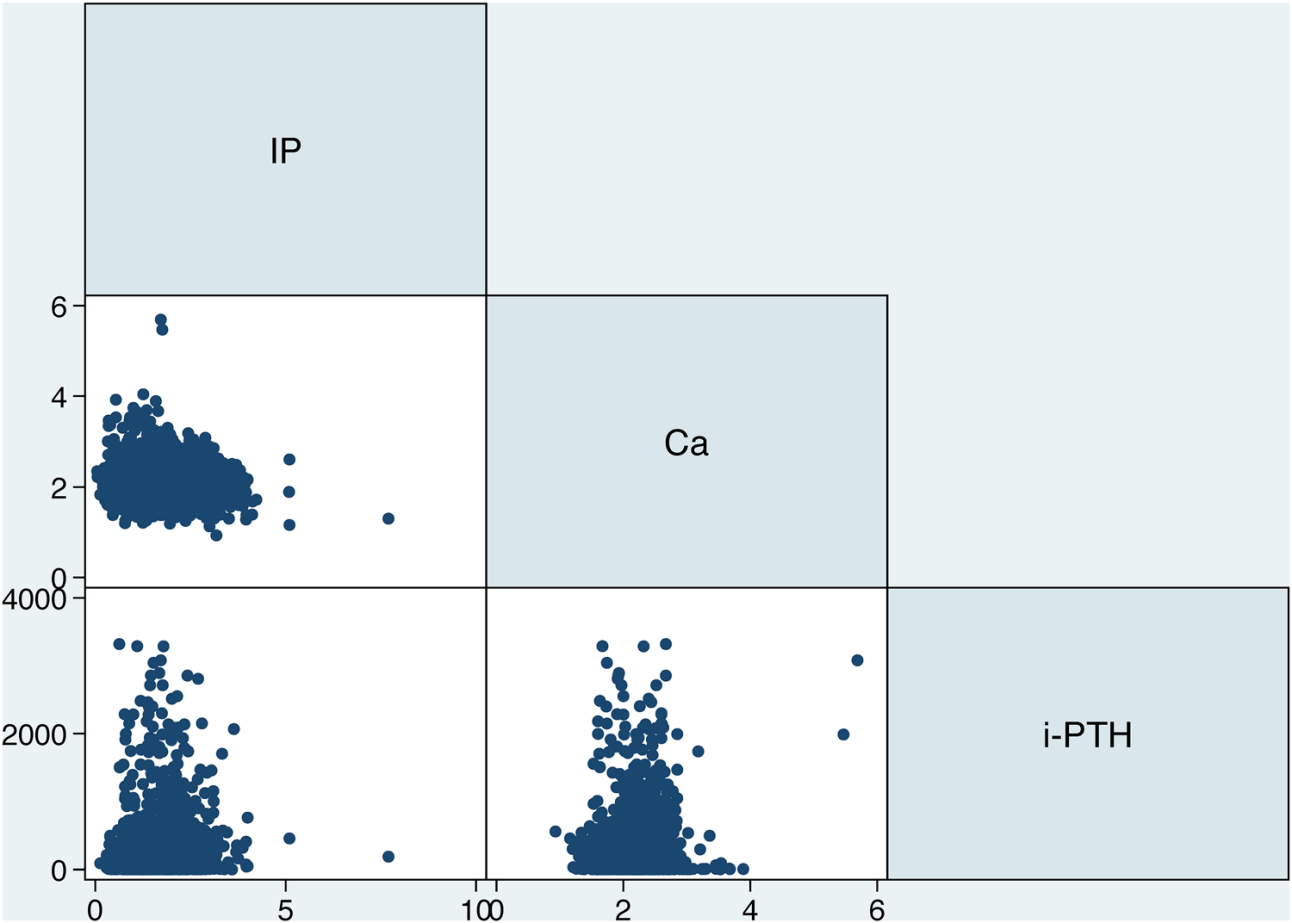
Correlation between serum inorganic phosphorus (IP), serum calcium (Ca), and intact parathyroid hormone (i-PTH) among all study subjects

The correlation between serum calcium, inorganic phosphorus, and intact parathyroid hormone in all study subjects is presented in Fig. 2. The correlation coefficients of three mineral metabolism markers were relatively small, and so all three mineral metabolism markers were retained in the following analyses.

### Overall risk prediction for CKD

Table 2 shows the risk prediction of mineral metabolism markers under study for CKD at different stages after propensity score weighting and adjustment for confounding factors. After applying Bonferroni correction to account for multiple testing with respect to 16 comparisons, statistical significance was recorded if the probability is less than 0.05/16, viz. 0.003. Per 0.5 mg/dL increment in serum calcium was significantly associated with 28–45% reduced risk of CKD at all stages. For inorganic phosphorus, per 0.5 mg/dL increment was significantly associated with 1.33-, 1.61-, and 2.85-fold increased risk of CKD at stages 1–2, 4, and 5, respectively. For calcium-phosphorus product, per 5 mg^2^/dL^2^ increment was significantly associated with 1.28- and 2.19-fold increased risk of CKD at stages 4 and 5, respectively. Regarding intact parathyroid hormone, significance was only noted for stage 5.

**Table 2:** Risk prediction of mineral metabolism markers for chronic kidney disease (CKD) at different stages in propensity score matching analysis

Significant risk factors	CKD stage 1–2	CKD stage 3	CKD stage 4	CKD stage 5
Ca (0.5 mg/dL increment)	0.66, 0.54 to 0.81, < 0.001	0.61, 0.49 to 0.77, < 0.001	0.55, 0.44 to 0.69, < 0.001	0.72, 0.61 to 0.86, < 0.001
IP (0.5 mg/dL increment)	1.33, 1.14 to 1.55, < 0.001	0.97, 0.81 to 1.16, 0.748	1.61, 1.35 to 1.91, < 0.001	2.85, 2.21 to 3.68, < 0.001
Ca × IP (5 mg^2^/dL^2^ increment)	1.07, 0.96 to 1.21, 0.227	0.97, 0.83 to 1.12, 0.660	1.28, 1.09 to 1.38, 0.001	2.19, 1.82 to 2.62, < 0.001
i-PTH (8 pg/mL increment)	1.06, 1.00 to 1.13, 0.040	1.01, 0.97 to 1.05, 0.548	1.10, 1.03 to 1.17, 0.004	1.09, 1.04 to 1.14, < 0.001

*Ca* serum calcium, *IP* inorganic phosphorus, *i-PTH* intact parathyroid hormone, *Ca × IP* calcium-phosphorus product

Data are expressed as odds ratio, 95% confidence interval, *P* value

### Subsidiary risk prediction for CKD

Table 3 shows the risk prediction of mineral metabolism markers under study for CKD at different stages according to age, sex, hypertension, and diabetes mellitus, respectively.

**Table 3:** Risk prediction of mineral metabolism markers for chronic kidney disease (CKD) at different stages in subsidiary analyses

Significant risk factors	CKD stage 1–2	CKD stage 3	CKD stage 4	CKD stage 5
Age < 60 years				
Ca (0.5 mg/dL increment)	0.67 0.55 to 0.83, < 0.001	0.74, 0.61 to 0.91, 0.004	0.77, 0.68 to 0.87, < 0.001	0.89, 0.84 to 0.95, 0.001
IP (0.5 mg/dL increment)	1.18, 1.04 to 1.34, 0.011	0.98, 0.84 to 1.15, 0.846	1.24, 1.12 to 1.37, < 0.001	1.58, 1.44 to 1.73, < 0.001
Ca × IP (5 mg^2^/dL^2^ increment)	1.09, 0.95 to 1.25, 0.202	0.88, 0.73 to 1.05, 0.145	1.14, 1.02 to 1.28, 0.016	1.47, 1.34 to 1.62, < 0.001
i-PTH (8 pg/mL increment)	1.24, 1.06 to 1.45, 0.006	1.75, 1.10 to 2.77, 0.017	1.25, 1.09 to 1.44, 0.001	1.13, 1.05 to 1.21, 0.001
Age > 60 years				
Ca (0.5 mg/dL increment)	0.76, 0.67 to 0.85, < 0.001	0.83, 0.75 to 0.91, < 0.001	0.85, 0.79 to 0.92, < 0.001	0.87, 0.81 to 0.94, < 0.001
IP (0.5 mg/dL increment)	1.00, 0.92 to 1.09, 0.938	1.03, 0.95 to 1.11, 0.439	1.31, 1.20 to 1.43, < 0.001	1.61, 1.49 to 1.75, < 0.001
Ca × IP (5 mg^2^/dL^2^ increment)	0.93, 0.84 to 1.02, 0.120	0.99, 0.89 to 1.05, 0.458	1.23, 1.13 to 1.35, < 0.001	1.58, 1.45 to 1.71, < 0.001
i-PTH (8 pg/mL increment)	1.20, 1.04 to 1.39, 0.011	1.25,1.05 to 1.49, 0.013	1.24, 1.10 to 1.41, < 0.001	1.18, 1.09 to 1.29, < 0.001
Men				
Ca (0.5 mg/dL increment)	0.68, 0.58 to 0.81, < 0.001	0.70, 0.58 to 0.85, < 0.001	0.56, 0.46 to 0.67, < 0.001	0.76, 0.66 to 0.88, < 0.001
IP (0.5 mg/dL increment)	1.14, 1.01 to 1.27, 0.027	1.16, 1.00 to 1.35, 0.043	1.42, 1.24 to 1.63, < 0.001	2.65, 2.15 to 3.27, < 0.001
Ca × IP (5mg^2^/dL^2^ increment)	1.05, 0.93 to 1.19, 0.434	1.04, 0.89 to 1.22, 0.620	1.28, 1.12 to 1.46, < 0.001	2.34, 1.93 to 2.83, < 0.001
i-PTH (8 pg/mL increment)	1.56, 1.22 to 1.99, < 0.001	1.42, 1.05 to 1.90, 0.021	1.72, 1.33 to 2.22, < 0.001	1.29, 1.13 to 1.46, < 0.001
Women				
Ca (0.5 mg/dL increment)	0.42, 0.26 to 0.67, < 0.001	0.61, 0.44 to 0.84, 0.002	0.53, 0.37 to 0.77, 0.001	0.83, 0.63 to 1.08, 0.169
IP (0.5 mg/dL increment)	1.15, 0.92 to 1.44, 0.210	1.05, 0.91 to 1.23, 0.460	1.11, 0.95 to 1.29, 0.178	1.60, 1.22 to 2.08, 0.001
Ca × IP (5 mg^2^/dL^2^ increment)	1.00, 0.78 to 1.27, 0.990	1.00, 0.86 to 1.18, 0.966	1.04, 0.90 to 1.22, 0.565	1.51, 1.15 to 1.96, 0.002
i-PTH (8 pg/mL increment)	1.68, 0.67 to 4.25, 0.269	1.42, 0.67 to 3.04, 0.357	1.34, 0.87 to 2.07, 0.188	1.17, 0.85 to 1.61, 0.330
Hypertension				
Ca (0.5 mg/dL increment)	0.61, 0.48 to 0.78, < 0.001	0.59, 0.46 to 0.75, < 0.001	0.58, 0.46 to 0.73, < 0.001	0.73, 0.61 to 0.88, 0.001
IP (0.5 mg/dL increment)	1.49, 1.26 to 1.77, < 0.001	1.08, 0.95 to 1.23, 0.222	1.29, 1.11 to 1.50, 0.001	3.13, 2.35 to 4.19, < 0.001
Ca × IP (5mg^2^/dL^2^ increment)	1.37, 1.15 to 1.61, < 0.001	1.00, 0.86 to 1.17, 0.951	1.19, 1.03 to 1.39, 0.022	2.56, 2.00 to 3.27, < 0.001
i-PTH (8 pg/mL increment)	1.45, 1.13 to 1.85, 0.003	1.69, 1.08 to 2.65, 0.022	1.32, 1.10 to 1.59, 0.003	1.29, 1.12 to 1.50, 0.001
Without hypertension				
Ca (0.5 mg/dL increment)	0.54, 0.39 to 0.75, < 0.001	0.92, 0.73 t0 1.16, 0.484	0.63, 0.48 to 0.83, 0.001	0.66, 0.48 to 0.90, 0.008
IP (0.5 mg/dL increment)	0.62, 0.46 to 0.85, 0.002	1.03, 0.84 to 1.26, 0.789	1.41, 1.11 to 1.79, 0.005	1.69, 1.28 to 2.25, < 0.001
Ca × IP (5 mg^2^/dL^2^ increment)	0.54, 0.40 to 0.75, < 0.001	0.99, 0.79 to 1.23, 0.934	1.21, 0.94 to 1.55, 0.133	1.44, 1.11 to 1.86, 0.005
i-PTH (8 pg/mL increment)	1.31, 0.93 to 1.85, 0.129	1.21, 0.93 to 1.56, 0.153	1.19, 0.99 to 1.48, 0.109	1.58, 0.96 to 2.59, 0.070
Diabetes mellitus				
Ca (0.5 mg/dL increment)	0.72, 0.54 to 0.96, 0.025	0.96, 0.74 to 1.24, 0.751	0.68, 0.53 to 0.86, 0.002	0.64, 0.44 to 0.91, 0.014
IP (0.5 mg/dL increment)	1.23, 1.01 to 1.49, 0.036	0.95, 0.73 to 1.23, 0.696	1.35, 1.12 to 1.63, 0.002	2.08, 1.46 to 2.97, < 0.001
Ca × IP (5 mg^2^/dL^2^ increment)	1.16, 0.96 to 1.41, 0.127	0.94, 0.73 to 1.22, 0.651	1.27, 1.06 to 1.51, 0.009	1.89, 1.35 to 2.62, < 0.001
i-PTH (8 pg/mL increment)	1.31, 1.06 to 1.64, 0.014	1.72, 1.12 to 2.65, 0.013	1.42, 1.14 to 1.77, 0.002	1.27, 1.03 to 1.57, 0.023
Without diabetes mellitus				
Ca (0.5 mg/dL increment)	0.57, 0.45 to 0.72, < 0.001	0.80, 0.65 to 0.99, 0.037	0.57, 0.44 to 0.73, < 0.001	0.78, 0.67 to 0.91, 0.002
IP (0.5 mg/dL increment)	1.13, 0.99 to 1.30, 0.069	1.07, 0.92 to 1.21, 0.380	1.31, 1.12 to 1.54, 0.001	2.57, 2.02 to 3.26, < 0.001
Ca × IP (5 mg^2^/dL^2^ increment)	1.03, 0.89 to 1.19, 0.706	0.98, 0.81 to 1.17, 0.814	1.19, 1.01 to 1.40, 0.032	2.33, 1.86 to 2.94, < 0.001
i-PTH (8 pg/mL increment)	1.23, 0.98 to 1.53, 0.069	1.23, 0.86 to 1.74, 0.245	1.21, 0.99 to 1.48, 0.061	1.17, 1.01 to 1.35, 0.039

*IP* inorganic phosphorus, *Ca* serum calcium, *i-PTH* intact parathyroid hormone, *Ca × IP* calcium-phosphorus product

Data are expressed as odds ratio, 95% confidence interval, *P* value

When study subjects were grouped by sex, the association between phosphorus and calcium–phosphorus product with stage 5 CKD was stronger in men than in women. Regarding intact parathyroid hormone, significance was noticed for the association with stage 5 in men, and there was no observable significance in women across all stages.

By hypertension, the prediction of mineral metabolism markers under study for CKD was significant at stages 1, 4 (except for calcium-phosphorus product), and 5. By diabetes mellitus, inorganic phosphorus and calcium-phosphorus product can predict the advanced stages of CKD, irrespective of having diabetes mellitus. Intact parathyroid hormone was a predictor for stage 4 CKD in diabetic patients, and no significance was noted in nondiabetic patients across all stages.

### Prediction of benefits from mineral metabolism markers

Table 4 shows the prediction accuracy gained by adding mineral metabolism markers to the basic model. From calibration aspect, reduction in both Akaike information criterion and Bayesian information criterion statistics was greater than 10 after adding serum calcium and intact parathyroid hormone to the basic model across all stages of CKD, and for inorganic phosphorus, reduction in both statistics was greater than 10 only at stages 4–5. Additionally, for stages 1–4, adding serum calcium or intact parathyroid hormone can significantly improve prediction performance, and for stage 5, significance was seen after adding any of three mineral metabolism markers, as reflected by likelihood ratio test, net reclassification improvement, and integrated discrimination improvement.

**Table 4:** Prediction accuracy gained by adding mineral metabolism markers to the basic model for chronic kidney disease (CKD) at different stages

Statistics	CKD stage 1–2				CKD stage 3			
	Basic model	Basic model plus Ca	Basic model plus IP	Basic model plus i-PTH	Basic model	Basic model plus Ca	Basic model plus IP	Basic model plus i-PTH
Calibration								
AIC	926.7	864.5	924.6	122.9	903.2	854.5	904.4	78.8
BIC	958.7	900.9	961.1	148.0	936.2	892.1	942.1	101.2
LR test (*χ*^*2*^)	Ref	61.02	0.35	17.1	Ref	48.7	0.8	27.5
LR test P	Ref	< 0.001	0.555	< 0.001	Ref	< 0.001	0.3750	< 0.001
Discrimination								
NRI (P)	Ref	< 0.001	0.4038	0.0015	Ref	0.0002	0.5418	0.0012
IDI (P)	Ref	< 0.001	0.7059	< 0.001	Ref	< 0.001	0.04376	< 0.001

Statistics	CKD stage 4				CKD stage 5			
	Basic model	Basic model plus Ca	Basic model plus IP	Basic model plus i-PTH	Basic model	Basic model plus Ca	Basic model plus IP	Basic model plus i-PTH
Calibration								
AIC	1160.3	1108.3	1151.7	169.15	1525.2	1491.5	1310.2	204.4
BIC	1197.4	1150.7	1194.1	202.74	1567.9	1540.3	1359.1	249.1
LR test (*χ*^*2*^)	Ref	52.9	10.5	33.1	Ref	35.2	216.7	63.9
LR test P	Ref	< 0.001	0.001	< 0.001	Ref	< 0.001	< 0.001	< 0.001
Discrimination								
NRI (P)	Ref	< 0.001	0.0441	0.0030	Ref	0.7340	< 0.001	0.0156
IDI (P)	Ref	< 0.001	0.0154	< 0.001	Ref	< 0.001	< 0.001	< 0.001

*AIC* Akaike information criterion, *BIC* Bayesian information criterion, *LR* likelihood ratio, *NRI* net reclassification improvement, *IDI* integrated discrimination improvement, *IP* inorganic phosphorus, *Ca* serum calcium, *i-PTH* intact parathyroid hormone, *Ref.* reference group

Basic model included age, sex, hypertension, diabetes mellitus, triglycerides, total cholesterol, high-density lipoprotein cholesterol, and uric acid

### Nomogram prediction model

**Fig. 3:**
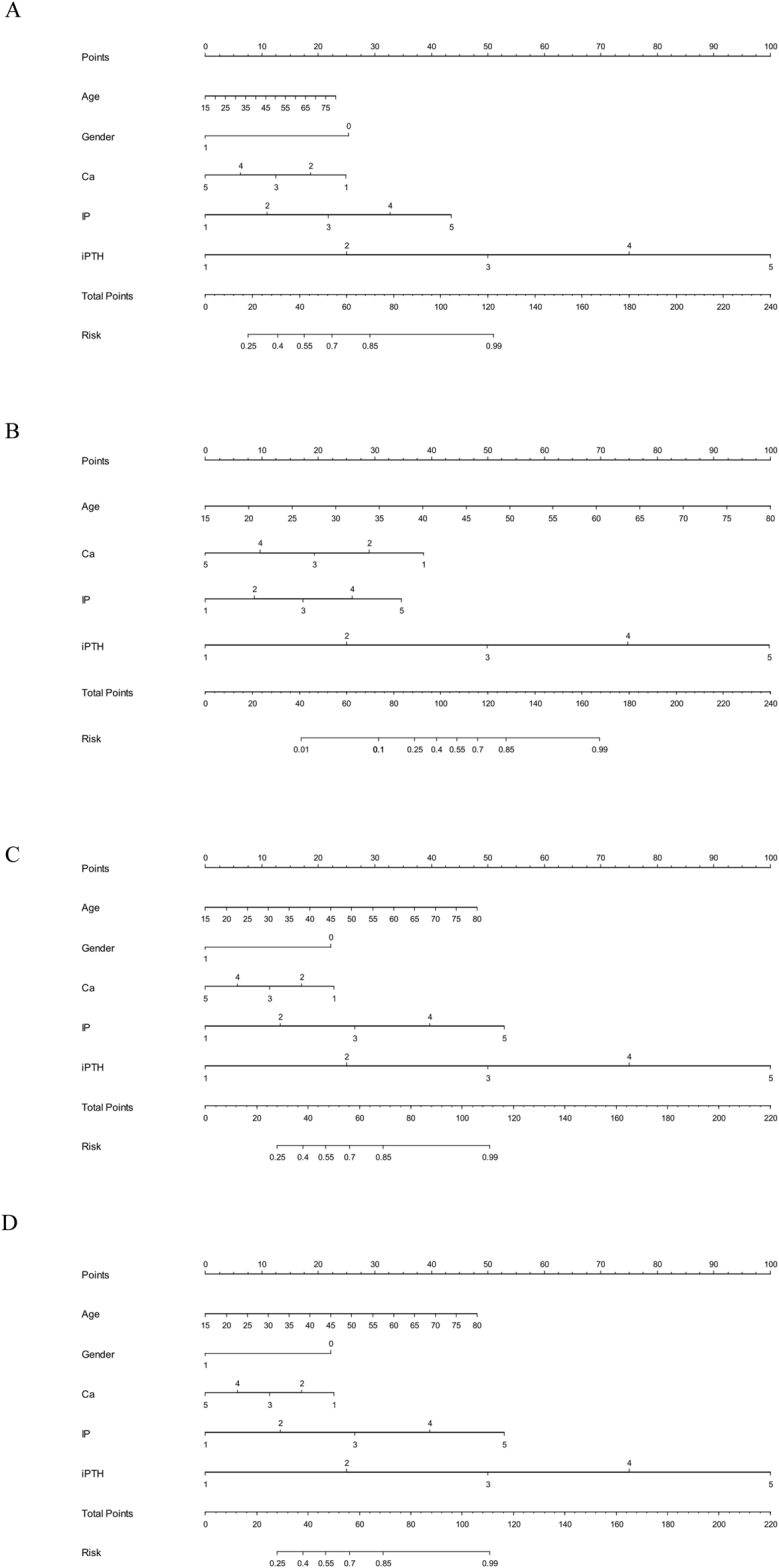
Nomogram prediction models on the basis of age, sex, and three mineral metabolism markers (serum inorganic phosphorus (IP), serum calcium (Ca), and intact parathyroid hormone (i-PTH)) for stage 1–2 (panel A), 3 (panel B), 4 (panel C), and 5 (panel D) chronic kidney disease, respectively. Sex was not incorporated in the nomogram for stage 3 due to nonsignificant effect

To facilitate clinical application, a nomogram prediction model was constructed on the basis of age, sex, and three mineral metabolism markers for stage 1–2, 3, 4, and 5 CKD, respectively (Fig. 3). The predictive accuracy and discriminative capability of all nomogram prediction models were assessed by concordance index, which was over 80% across all stages, indicating significant improvement in model performance.

Taking stage 4 as an example, assuming a men aged 45 years old (20 points) with serum calcium of 8 mg/dL (25 points), inorganic phosphorus of 6 mg/dL (42 points), intact parathyroid hormone of 100 pg/mL (20 points), the probability of having stage 4 CKD was estimated to be 90%.

## Discussion

The aim of this retrospective study was to examine the association of three mineral metabolism markers with risk of different stages of CKD among Chinese adults. The key finding is that elevated concentrations of serum calcium were associated with reduced risk of all-stage CKD, whereas that of inorganic phosphorus, calcium-phosphorus product, and intact parathyroid hormone increased the risk of only advanced stages. Moreover, the risk prediction of these markers was more evident in males and hypertensive subjects. Our findings suggest that mineral metabolism markers may help identify subgroups at high risk of having CKD or advancing to advanced stages.

Our findings are biologically plausible. Growing evidence indicates that calcium and phosphorus homeostasis plays an important role in the pathophysiology of CKD. Some researchers reported that increased parathyroid hormone occurred before abnormalities in serum calcium and phosphate concentrations [29], and it can maintain serum calcium within normal physiological ranges via increasing calcium efflux from bone, renal calcium reabsorption, and phosphate excretion. Moreover, increased secretion of parathyroid hormone can stimulate renal phosphate excretion in CKD [30], and parathyroid hormone secretion increased in response to changes in calcium and phosphate [29]. Based on above evidence, it is reasonable to speculate that abnormalities in mineral homeostasis are implicated in the development and progression of CKD.

Our findings reinforced this speculation by demonstrating that serum calcium was a predictive factor for all-stage CKD, yet inorganic phosphorus, calcium-phosphorus product, and intact parathyroid hormone were risk factors for its advanced stages. The study by Rouached and colleagues showed that high intact parathyroid hormone may less likely happen with estimated glomerular filtration rate greater than 80 ml/min, and serum calcium and inorganic phosphorus can be maintained in normal ranges until estimated glomerular filtration rate is less than 40 ml/min [29]. Extending the results of this study [29], our analysis revealed that serum calcium concentrations were significantly lower in patients at all stages, indicating that serum calcium is a robust indicator of CKD.

It is widely recognized early monitoring of serum mineral metabolism markers is important for public health planning. Currently, most studies examining mineral metabolism markers and CKD are restricted to advanced stages [31, 32]. Although the 2017 KDIGO guidelines of CKD-MBD have recommended monitoring serum concentrations of calcium, phosphorus, and intact parathyroid hormone beginning in stage 3 CKD, our subsidiary analyses indicated that the risk prediction of these markers hinged upon sex and hypertension status. For instance, intact parathyroid hormone was associated with the significantly increased risk of stages 1–2 and 4–5 CKD in men or hypertensive subjects, whereas this association remained nonsignificant in women or normotensive subjects. Additionally, Bellasi and colleagues in a prospective study reported that the magnitude of risk associated with hyperphosphatemia varied depending on age, sex, diabetes, and different stages of CKD [33], which can consolidate the subsidiary results of the present study. Further, another cohort study by De Boer and colleagues demonstrated that the association between parathyroid hormone and chronic renal insufficiency differed across races. In view of this racial-dependent assocaition, we agree that it is of importance to establish data on the risk prediction of mineral metabolism markers for the risk and severity of CKD in each racial or ethnical group.

What’s more, we constructed nomogram models for different stages of CKD after propensity score matching analysis, with decent prediction accuracy. For practical reasons, these nomogram models can be used in routine clinical practice to facilitate clinical decision-making and the personalized management of CKD.

Several possible limitations should be acknowledged for this study. First, the present analysis was based on cross-sectional data, which will preclude further comments on causality inference, especially the trajectory of CKD progression. Second, some unmeasured characteristics of study subjects such as smoking, drinking, dietary habits, as well as drug regimens such as RAS inhibitors and/or statins might confound the association of mineral metabolism markers with CKD [34]. Third, all study subjects were recruited from a mono center, which might raise a possibility of population stratification.

Despite these limitations, our findings indicate that serum calcium was associated with the risk of all-stage CKD, whereas the association for inorganic phosphorus, calcium-phosphorus product, and intact parathyroid hormone was significant at advanced stages. Importantly, the prediction capability of these markers was more evident in men and hypertensive subjects. Hence, more attention should be given to the balance of mineral metabolism markers even in patients with early stages of CKD to control and prevent the development and progression. We also agree that further investigations on the molecular mechanisms linking mineral metabolism markers and CKD are warranted.
